# A Multicenter, Double‐Blind, Placebo‐Controlled, Randomized Clinical Trial of Oral Anticoagulation in Systemic Sclerosis‐Related Pulmonary Arterial Hypertension—Results From the SPHInX Study

**DOI:** 10.1002/pul2.70331

**Published:** 2026-06-08

**Authors:** Mandana Nikpour, Alicia Calderone, Susanna M. Proudman, Dylan Hansen, Eli Gabbay, Nathan Dwyer, Gregory Keir, Vivek Thakkar, Peter K. K. Wong, Anne Keogh, Andrew Burns, David Prior, Harshal Nandurkar, David S. Celermajer, Joanne Sahhar, Rachelle Buchbinder, Wendy Stevens

**Affiliations:** ^1^ The University of Sydney School of Public Health Sydney New South Wales Australia; ^2^ Sydney Musculoskeletal Research Flagship Centre Camperdown New South Wales Australia; ^3^ Department of Rheumatology Royal Prince Alfred Hospital Camperdown New South Wales Australia; ^4^ Department of Medicine at St. Vincent's Hospital University of Melbourne Fitzroy Victoria Australia; ^5^ Department of Rheumatology St. Vincent's Hospital Melbourne Fitzroy Victoria Australia; ^6^ Adelaide Medical School University of Adelaide Adelaide South Australia Australia; ^7^ Rheumatology Unit Royal Adelaide Hospital Adelaide South Australia Australia; ^8^ Respiratory Medicine Clinic St John of God Subiaco Hospital Subiaco Western Australia Australia; ^9^ School of Medicine University of Notre Dame Australia Fremantle Western Australia Australia; ^10^ Institute for Respiratory Health University of Western Australia Nedlands Western Australia Australia; ^11^ Department of Cardiology Royal Hobart Hospital Hobart Tasmania Australia; ^12^ Tasmanian School of Medicine University of Tasmania Hobart Tasmania Australia; ^13^ Department of Respiratory and Sleep Medicine Princess Alexandra Hospital Woolloongabba Queensland Australia; ^14^ Lung and Allergy Research Centre University of Queensland Brisbane Queensland Australia; ^15^ Department of Rheumatology Liverpool Hospital Liverpool New South Wales Australia; ^16^ Macquarie Medical School Macquarie University Sydney New South Wales Australia; ^17^ Department of Rheumatology Westmead Hospital Westmead New South Wales Australia; ^18^ Sydney Medical School University of Sydney Camperdown New South Wales Australia; ^19^ Rural Clinical School University of New South Wales Coffs Harbour New South Wales Australia; ^20^ Heart Transplant Unit, St Vincent's Hospital Sydney Darlinghurst New South Wales Australia; ^21^ School of Clinical Medicine University of New South Wales Darlinghurst New South Wales Australia; ^22^ Department of Cardiology St. Vincent's Hospital Melbourne Fitzroy Victoria Australia; ^23^ Albury Wodonga Health Albury New South Wales Australia; ^24^ Clinical Haematology, Alfred Health Melbourne Victoria Australia; ^25^ Australian Centre for Blood Diseases, Alfred Monash Research and Education Precinct Monash University South Yarra Victoria Australia; ^26^ Department of Cardiology Royal Prince Alfred Hospital Camperdown New South Wales Australia; ^27^ Heart Research Institute Newtown New South Wales Australia; ^28^ Department of Rheumatology Monash Health Clayton Victoria Australia; ^29^ Department of Medicine Monash University Clayton Victoria Australia; ^30^ Musculoskeletal Health and Wiser Health Care Units, School of Public Health and Preventive Medicine Monash University Melbourne Victoria Australia

**Keywords:** autoimmune, connective tissue disease, therapeutics

## Abstract

The SPHInX study is the first‐ever randomized controlled trial (RCT) seeking to inform an area of equipoise regarding the efficacy of oral anticoagulation as adjunct therapy in systemic sclerosis‐related pulmonary arterial hypertension (SSc‐PAH). The SPHInX study was an Australian multicenter, double‐blind Phase III RCT of 1:1 oral apixaban 2.5 mg twice daily versus placebo over 3 years, as additional therapy in patients with SSc‐PAH. The primary end‐point was time to clinical worsening (TtCW), or death. A sample size of 85 per arm was required to show a two‐fold reduction in TtCW. Participants experiencing clinical worsening events continued in the study, allowing for assessment of exploratory endpoints (including physical function and quality of life measures) up until 3 years of treatment. Among 11 SSc‐PAH participants assigned apixaban treatment and 14 participants assigned placebo, apixaban demonstrated no benefit compared to placebo for TtCW from commencement until 30 days after discontinuation of study drug, Cox proportional hazard ratio 0.92 (95% confidence interval 0.32–2.66), *p* = 0.88. There was no difference between treatment groups in event‐free survival, all‐cause mortality, or exploratory endpoints. New iron deficiency anemia occurred in 9 (81.8%) apixaban treated participants compared to 4 (28.6%) controls. Although strict selection criteria in a complex disease meant recruitment was insufficient for the primary efficacy endpoint, the SPHInX RCT showed no signal for benefit with anticoagulation as adjunct therapy, with a high frequency of iron deficiency anemia suggesting that even at low doses, risk may outweigh benefit with anticoagulation in SSc‐PAH.

**Trial Registration:** Australian New Zealand Clinical Trials Registry Registration Number: ACTRN12614000418673 (http://www.ANZCTR.org.au/ACTRN12614000418673.aspx).

## 1 Introduction

Systemic sclerosis (SSc) is an autoimmune connective tissue disease (CTD) that, although rare, owing to its chronicity and multi‐organ manifestations, carries the greatest burden of case‐based mortality and morbidity among the rheumatological diseases [1, 2, 3, 4, 5]. Pulmonary arterial hypertension (PAH) is one of the most life‐threatening vascular complications of SSc, accounting for up to 30%–40% of all deaths in SSc [6, 7, 8, 9]. Untreated, SSc‐related PAH (SSc‐PAH) may have a rapidly fatal course, with death resulting from right ventricular failure and arrhythmias [10].

Although advanced pulmonary vasodilator therapies have become increasingly available and confer a survival benefit in PAH, SSc‐PAH patients continue to display a worse prognosis than patients with idiopathic PAH (iPAH) [11, 12]. Additionally, combination therapy with more than one agent, to target multiple pathogenic pathways, yields demonstrable survival advantages [13, 14]. Hence, further research into the pathophysiology and possible therapeutic targets is required.

In situ thrombosis is a likely contributor to the pathophysiology of SSc‐PAH, with pulmonary vascular (venous and arterial) thrombosis appearing as a common histological feature in both iPAH and CTD‐PAH tissue specimens [15, 16, 17]. While several observational studies have suggested a survival benefit with anticoagulation in PAH, other observational studies have not supported this finding [14, 18, 19, 20, 21, 22]. Moreover, there is great variability between rheumatologists versus respiratory physicians in beliefs and prescribing habits regarding anticoagulation as adjunct therapy in PAH [23, 24]. The lack of convincing evidence surrounding the benefit of anticoagulation in this condition, and conflicting outcomes emerging between the largest observational studies to date [25, 26, 27], demands resolution of this contentious issue through the gold standard approach of a randomized controlled trial (RCT).

We hypothesized that anticoagulation prolongs survival, increases functional capacity and overall wellbeing, and reduces hospitalizations in patients with SSc‐PAH. The detailed rationale and study design of this first of its kind RCT has been previously published [20, 28]. The aim of the Scleroderma‐Pulmonary Arterial Hypertension Itervention with Apixaban (SPHInX) study was to evaluate the efficacy and safety of treatment with the oral anticoagulant apixaban (a factor Xa inhibitor) in SSc‐PAH, by undertaking a multicenter, double‐blind, placebo‐controlled RCT. Exclusion criteria were justifiably strict to ensure the safety of participants at increased risk of bleeding events [20, 29, 30, 31], however, they had a negative impact on recruitment of the target sample size. In this report we describe the updated methodology that was employed attempting to boost sample size, and we present the study outcomes.

## 2 Methods

### 2.1 Study Design

The SPHInX study was an investigator‐initiated and led Australian multicenter, randomized, double‐blinded, placebo‐controlled, Phase III clinical trial of apixaban 2.5 mg twice daily (bid) versus placebo treatment over 3 years, as additional therapy in SSc‐PAH patients already receiving advanced pulmonary vasodilators as standard of care [28]. The SPHInX study was registered prospectively on April 16, 2014 with the Australian New Zealand Clinical Trials Registry (ACTRN12614000418673).

### 2.2 Ethical Approval

Institutional Review Board approval was granted by the Human Research Ethics Committees of St. Vincent's Hospital Melbourne, Royal Perth Hospital, University of Western Australia, and Menzies Research Institute of Tasmania. All procedures followed were in accordance with institutional guidelines, acknowledged by the Governance offices of all hospitals involved in the trial (Fiona Stanley Hospital, Gold Coast University Hospital, Liverpool Hospital, Monash Health, Princess Alexandra Hospital, Royal Adelaide Hospital, Royal Hobart Hospital, Royal Prince Alfred Hospital, St Vincent's Hospital Sydney, The Alfred Hospital, and The Queen Elizabeth Hospital).

### 2.3 Study Population

The RCT was undertaken at 13 Australian PAH treatment centers across 6 states (New South Wales, Queensland, South Australia, Tasmania, Victoria, Western Australia). During the course of routine outpatient care at these centers, cardiologists, respirologists, and rheumatologists assessed patients for eligibility. Between September 2014 and February 2019, eligible patients meeting all inclusion criteria and none of the exclusion criteria in Table 1, provided written informed consent prior to study enrollment and treatment allocation, following adequate explanation of the aims, methods, objectives, and potential hazards of the trial by the responsible investigator.

**Table 1:** Eligibility criteria of the SPHInX study.

Inclusion criteria^a^	Exclusion criteria^b^
1. Male and female patients aged from 18 to 75 years inclusive. 2. Patients with symptomatic permissible Group 1 pulmonary hypertension subcategories: i. Idiopathic PAH ii. Heritable PAH, including genetic mutations in BMPR2, ALK‐1, ENG, SMAD9, CAV1, KCNK3, or unknown iii. Associated with CTD such as systemic sclerosis defined by the ACR/EULAR criteria for SSc. 3. RHC at any time prior to Baseline demonstrating the following hemodynamic characteristics in line with current international guidelines for diagnosis of PAH: i. resting mPAP ≥ 25 mmHg, and ii. resting PVR ≥ 3 woods units, and iii. resting PAWP or LVEDP ≤ 15 mmHg, or iv. if PVR cannot or has not been measured, then mPAP ≥ 30 mmHg with PAWP or LVEDP ≤ 15 mmHg. 4. Six minute walk distance greater than 50 meters at screening and/or baseline. 5. Other causes of PAH, in particular CTEPH must have been previously excluded by either a V/Q scan or CTPA. 6. Currently taking at least one of the ETRA or PDE‐5 inhibitor medications in a stable dose for the 2 months prior to baseline (either bosentan, ambrisentan or macitentan, and/or sildenafil or tadalafil). 7. Female participants of childbearing potential must test negative for pregnancy. 8. Male and female participants of childbearing potential (biologically capable of having children and sexually active) must agree to use a highly effective method of contraception throughout the study and for at least 28 days after the last dose of the study drug. 9. Female participants who are not of childbearing potential must meet at least one of the following criteria: i. have undergone documented hysterectomy and/or bilateral oophorectomy, ii. have medically confirmed ovarian failure, or iii. achieved postmenopausal status, defined as cessation of regular menses for at least 12 consecutive months with no alternative pathological or physiological cause, and iv. have a serum follicle‐stimulating hormone level within the laboratory's reference range for postmenopausal females.	1. Pulmonary hypertension due to any other cause than idiopathic, heritable, or CTD‐PAH. 2. Moderate or severe obstructive lung disease, i.e., FEV1/FVC ratio < 70% and FEV1 < 65% of predicted value after bronchodilator administration. 3. Moderate or severe restrictive lung disease, i.e., FVC < 70% of predicted value, provided that HRCT scan demonstrates moderate to severe changes of ILD, or FVC < 60% of predicted value, regardless of HRCT result. 4. Moderate or severe hepatic impairment (i.e., Child‐Pugh Class B or C). 5. Documented left ventricular dysfunction (i.e., ejection fraction < 45%). 6. Severe renal insufficiency (estimated creatinine clearance < 25 mL/min, or serum creatinine > 200 µmol/L). 7. Receiving any investigational drugs within 1 month prior to, or at baseline. 8. Receiving continuous intravenous epoprostenol or iloprost at baseline or have planned to initiate this therapy within the next 3 months. 9. Psychotic, addictive, or other disorder limiting the ability to provide informed consent or to comply with study requirements. 10. Life expectancy due to another condition of less than 12 months. 11. Females who are breastfeeding or pregnant (positive pre‐randomization serum pregnancy test) or plan to become pregnant during the study. 12. Known hypersensitivity to drugs of the same class as the study drug, or any of the excipients of the drug formulations. 13. Gastrointestinal tract bleeding in the last 12 months due to GAVE or unexplained iron deficiency anemia (in the last 12 months). 14. Hemoglobin < 100 g/L at screening. 15. Significant falls risk. 16. Taken oral or subcutaneous anticoagulants (e.g., warfarin, apixaban, rivaroxaban, dabigatran, enoxaparin, dalteparin, or heparin) for more than 3 months since the diagnosis of PAH. 17. Prosthetic valve in situ. 18. Currently in atrial fibrillation. 19. Not on either an ETRA or PDE‐5 inhibitor. 20. Known bleeding disorders and/or platelet count < 100 at screening and/or INR > 1.2 at screening. 21. Brain, spinal, or eye surgery within the last 1 month. 22. Uncontrolled systemic hypertension defined as either systolic blood pressure > 179 mmHg or diastolic blood pressure > 109 mmHg at screening. 23. Documented episode of either pulmonary embolus or deep venous thrombosis since diagnosis of PAH. 24. Current, or active in the last 1 month, major bleed that is life threatening, causes chronic sequelae or consumes major healthcare resources [30].

Abbreviations: ACR = American College of Rheumatology, CTD = connective tissue disease, CTD‐PAH = connective tissue disease associated pulmonary arterial hypertension, CTEPH = chronic thromboembolic pulmonary hypertension, CTPA = computed tomography pulmonary angiogram, EULAR = European League Against Rheumatism, ETRA = endothelin‐1 receptor antagonist, FEV1 = forced expiratory volume in one second, FVC = forced vital capacity, GAVE = gastric antral vascular ectasiae, HRCT = high resolution computed tomography, ILD = interstitial lung disease, INR = international normalization ratio, LVEDP = left ventricular end diastolic pressure, mPAP = mean pulmonary arterial pressure, PAH = pulmonary arterial hypertension, PAWP = pulmonary arterial wedge pressure, PDE‐5 = phosphodiesterase type 5, PVR = pulmonary vascular resistance, V/Q = ventilation/perfusion, RHC = right heart catheterization.

a All items must be present for eligibility into the clinical trial.

b Participants must not meet any of the exclusion criteria for eligibility into the clinical trial.

During the first 24 months of enrollment, eligibility was limited to adults with symptomatic SSc‐PAH as defined by the American College of Rheumatology/European League Against Rheumatism 2013 classification criteria for SSc [32], and international guidelines for diagnosis of PAH [33], diagnosed by right heart catheterization (RHC) less than 3 years prior to the baseline visit. However, slow recruitment necessitated expansion of the inclusion criteria that were previously described [28], to also include CTD‐PAH, heritable PAH (hPAH), and iPAH patients with prevalent disease, meeting the updated clinical classification of Group 1 pulmonary hypertension (PH) [34]. Despite falling short of the desired sample size of 85 per arm, recruitment was abandoned when the funding period ended.

### 2.4 Randomization and Masking

Randomization to placebo or study drug in a 1:1 ratio stratified according to study site was performed by a statistician who was not associated with any study site, using computer‐generated block randomization, and delivered in sealed opaque envelopes to site pharmacists. Participants were assigned to study treatment by site pharmacists, not involved in any other aspect of the trial, according to the site randomization sequence. Participants, healthcare providers, investigators, data collectors, and outcome assessors were blinded to treatment assignment. To ensure allocation concealment, the appearance of the investigational drug and its packaging were indistinguishable from the matching placebo, both manufactured by Bristol‐Myers Squibb Limited (BMS), New Jersey, USA.

A log of every access to individual participant unblinding codes was kept and all requests for unblinding were clearly justified by medical emergencies wherein management would be improved by knowledge of the blinded treatment assignment. The randomization code overall was not made available to investigators, including the study statistician, until after data analysis was complete.

### 2.5 Procedures

#### 2.5.1 Treatment Exposure and Compliance

The RCT intervention and study assessments were performed as described previously [28]. In brief, the study drug apixaban, BMS‐562247‐01 or matching placebo, was administered orally, in the form of 2.5 mg reddish brown, plain, oval‐shaped, shallow bi‐convex film‐coated tablets. The dose regimen was twice daily with an interval of approximately 12 h, over 36 months of treatment. Study drug interruption not exceeding 8 weeks, or premature discontinuation occurred when in the best interests of the participant (i.e., due to adverse event, diagnostic or therapeutic procedure, laboratory abnormalities, unblinding, or withdrawal of consent). Participants were asked to return all unused study drug at follow‐up visits and to self‐report any missed doses of study drug, and changes to prescribed concomitant medications. Study drug adherence was assessed by recording quantities of returned study drug at each follow‐up visit.

Adjunct therapy with at least one advanced pulmonary vasodilator from the categories of endothelin‐1 receptor antagonist (ETRA) and/or a phosphodiesterase type‐5 (PDE‐5) inhibitor, maintained at a stable dose for at least 2 months prior to baseline, and other permissible concomitant medications were continued throughout the study as standard of care practice. Concomitant use of noninterventional anticoagulation was specifically prohibited by the exclusion criteria but use of a single antiplatelet agent was allowed at physician discretion. Commencement of any new PAH‐specific treatment or a dose increase of such therapy during the study period was documented for adjudication by the blinded endpoint committee.

#### 2.5.2 Study Assessments

**Figure 1:**
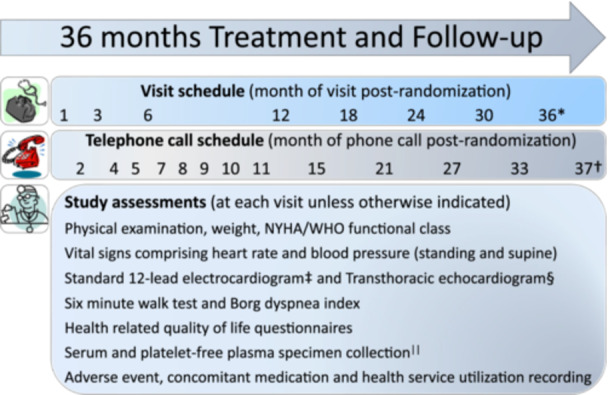
SPHINX study assessment timeline. Over the course of 36 months study treatment, participants visited study sites at the following times post‐randomization: 1, 3, 6, 12, 18, 24, 30, and 36 months (*end of study visit, performed on the day of permanent cessation of study drug, sooner than 36 months in exceptional circumstances). Telephone follow‐up maintained monthly contact between scheduled visits in the first 12 months, occurring third‐monthly thereafter, until 30 days after the end of study visit (^†^37 months post‐randomization at the latest), to capture self‐reported compliance and health service utilization, in particular changes to concomitant medication, adverse event surveillance, and clinical worsening events. ^‡^Performed at baseline, 6, and 24 months, clinical worsening event, and end of study visits. ^§^Data obtained within 2 months of baseline, 6, and 24 months visits. ^||^Storage at −80°C from baseline, 6, and 24 month visits, to be batch tested in a single laboratory at the end of study, for serum NT‐proBNP and plasma anti‐Factor Xa.

Complete medical history and data confirming presence of all inclusion criteria, and absence of all exclusion criteria (Table 1), were collected by the treating investigator at baseline, prior to commencement of study drug administration. Health‐related and safety outcomes were monitored by the treating investigator monthly in the first 12 months of the intervention, then third‐monthly for the remainder of the intervention period, ending 30 days after the permanent cessation of study drug (Figure 1). Additional interim follow‐up visits were conducted in case of suspected clinical worsening events (CWEs).

Functional capacity was assessed at every study visit using the modified New York Heart Association/World Health Organization (NYHA/WHO) functional class [35] and 6 min walk test performed in a standardized, nonencouraged fashion, measuring the walking distance covered by the participant during a 6 min period (6MWD) followed immediately by the Borg dyspnea index, which rates dyspnea severity on a visual analog scale from “0” to “10” [36]. Health‐related quality of life (HRQoL) was self‐reported by study participants independently completing the following validated questionnaires at every visit: The Medical Outcomes Study 36‐Item Short Form Health Survey (SF‐36) [37], the scleroderma‐modified Stanford Health Assessment Questionnaire (SHAQ) including the total Visual Analog Scale selections across 6 areas of SSc‐related limitations [38], and the mean of 8 category scores from the 20‐item Sanford Health Assessment Questionnaire (HAQ‐DI) [39], and the Cambridge Pulmonary Hypertension Outcome Review (CAMPHOR) [40].

#### 2.5.3 Adverse Event Monitoring

Treatment‐emergent adverse events (AEs), including abnormalities in routine laboratory results, were either spontaneously reported or elicited during open‐ended questioning of participants at all study visits up to 30 days post‐cessation of study drug intake. Investigators followed all reported AEs to resolution, reporting on the intensity of each AE, whether it was serious or nonserious, and whether there was any reasonable causal relationship between the AE and use of study drug. An independent Data and Safety Monitoring Board, comprising a rheumatologist, hematologist, cardiologist, and gastroenterologist, reviewed unblinded safety and tolerability data on seven occasions, to ensure safety of participants for the duration of the study.

At the conclusion of the study, while still blinded to treatment allocation, a panel of three study investigators reviewed all AEs to confirm their nature as a new medical occurrence or worsening of a pre‐existing condition, and to categorize free‐text narratives into common diagnostic terminology. For instance, the appearance in laboratory tests of low hemoglobin accompanied by reduced ferritin levels that were not present at enrollment was described as new iron deficiency anemia.

### 2.6 Outcomes

#### 2.6.1 Primary Outcome

A composite primary outcome was employed [41]. “Time to clinical worsening” (TtCW) was defined as time from randomization to the first adjudicated CWE, up until 30 days post‐cessation of study drug treatment. Within the composite primary endpoint CWEs were singular or combinations of requisite events including, all‐cause mortality, nonelective hospital stay for worsening of PAH (due to either initiation of intravenous prostenoids or chronic oxygen therapy, lung transplantation, or atrial septostomy), or disease progression defined by the combination of at least two of the following features, reduction from baseline in 6MWD by 15%, confirmed by two tests done within 2 weeks, plus worsening of functional class (except for patients already in Functional Class IV), or appearance of symptoms of right heart failure. At any visit where a study participant had a suspected CWE, the treating investigator reported on all measurable parameters that would support adjudication of the composite primary endpoint by the SPHInX study endpoint adjudication committee (comprised of at least three blinded investigators who were not treating the given participant).

#### 2.6.2 Exploratory Outcomes

When clinically appropriate, the study drug was continued in a blinded fashion following CWE, to enable quantification of the total number of CWEs during the study period as a secondary endpoint. Exploratory endpoints evaluated annually included all‐cause mortality (adjusted for time since diagnosis of PAH), absence of worsening in NYHA/WHO functional class, change in 6MWD, change in Borg dyspnea index, change in HRQoL, and number per year and in‐patient duration of all‐cause versus PAH‐related hospitalizations. All‐cause hospitalizations include elective and emergency presentations for any adverse events or pre‐existing conditions.

Safety and tolerability endpoints comprised treatment‐emergent adverse events (serious and nonserious) including marked laboratory abnormalities up to 7 days after last study drug intake, adverse events leading to premature discontinuation of study drug, initiation of new medications, health service utilization (general practitioner or specialist services), and change from baseline to end of study in vital signs.

### 2.7 Statistical Analysis

Study data were collected and managed using the secure, web‐based software platform REDCap [42, 43], hosted at St. Vincent's Hospital (Melbourne). Chief Investigator Nikpour had full access to all the data in the study and takes responsibility for its integrity and the data analysis. There were no interim analyses and randomization code was not broken until all primary and secondary endpoints had been reviewed and adjudicated. The data were then exported to STATA version 15.1 for analysis of the intention to treat SSc‐PAH participants.

The statistical analysis plan has been previously published [28]. A total sample size of 170 participants (85 per arm) was required to detect a two‐fold difference in hazard ratio for TtCW between placebo and apixaban treatment, with 80% power and 95% confidence. Kaplan–Meier curves and Cox proportional hazard ratio (95% confidence interval) were presented for time to event outcomes. A sensitivity analysis was conducted using time since diagnosis of PAH by RHC as covariate. In order to evaluate the robustness of results, the primary endpoint was also analyzed on the per‐protocol set, with 80% compliance as the cut‐off point. In addition to nonadherence to the study drug, participants without CWEs who permanently discontinued treatment prematurely within 876 days (< 80% of 3 years) were considered noncompliant and excluded from the per‐protocol set.

Comparisons between treatment groups for categorical variables were presented as frequency (percentage) with a *p* value calculated using Fisher's exact test or *χ* ^2^ test as appropriate. For normally distributed continuous variables, a mean (standard deviation) was presented with *p* value calculated by Student's *t*‐test; for non‐normally distributed continuous variables the median (interquartile range) were presented with *p* value calculated by the Wilcoxon rank‐sum test. Multilevel mixed‐effects linear regression models were used to assess repeated measures continuous outcomes with the predicted marginal means for placebo and apixaban treatment presented at each time point.

## 3 Results

### 3.1 Demographics

**Figure 2:**
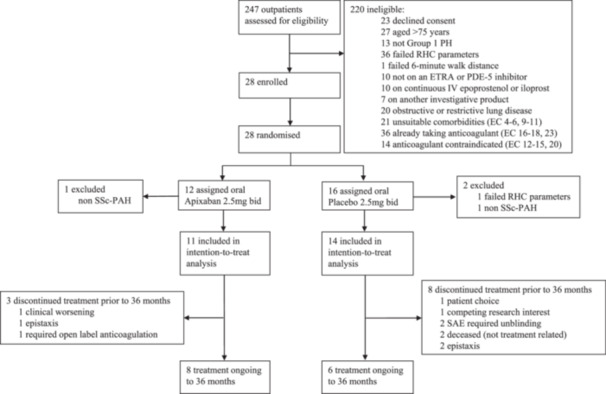
Diagram depicting the flow of participants through each stage of the SPHInX study. bid = twice daily oral intake, EC = exclusion criteria, ETRA = endothelin‐1 receptor antagonist, IV = intravenous, PDE‐5 = phosphodiesterase type 5, PH = pulmonary hypertension, RHC = right heart catheterization, SAE = serious adverse event, SSc‐PAH = systemic sclerosis related pulmonary arterial hypertension.

Participants were recruited between September 15, 2014 and February 22, 2019. Figure 2 depicts the progress of participants from screening through to analysis. Among 247 outpatients assessed for eligibility at cardiology, respiratory, and rheumatology departments of all approved study centers, 28 participants were randomized (12 apixaban and 16 placebo) from 7 study centers Australia‐wide (Adelaide, Brisbane, Coffs Harbour, Hobart, Melbourne, Perth, Sydney). The most common reasons for exclusion among the 220 ineligible patients were 36 (14.6%) failed to meet RHC parameters for diagnosis of PAH (including one inadvertently randomized to placebo and withdrawn within 30 days of treatment), and 36 (14.6%) were already being anticoagulated. Anticoagulation was contraindicated in 14 (5.7%) of the screened outpatients, mainly due to a history of unexplained iron deficiency anemia in the previous 12 months.

Of all eligible Group 1 PH participants randomized, one assigned to apixaban with hPAH and one assigned to placebo with iPAH were excluded from the intention to treat analysis because there were too few such participants to allow for meaningful subgroup analyses. Baseline characteristics of the SSc‐PAH participants treated (11 apixaban, 14 placebo) are presented in Table 2. Study participants were predominantly Caucasian postmenopausal females, with SSc‐PAH of median (±IQR) duration 1.27 (0.41–1.97) years overall. The complete data for underlying SSc features and comorbidities at baseline are provided in Supporting Information S1: Tables 1 and 2, respectively. All participants were prescribed an ETRA as adjunct PAH therapy at baseline, commonly combined as dual PAH therapy with a PDE‐5 Inhibitor in three apixaban and eight placebo participants. Over half of the cohort also took antihypertensive vasodilators and oral diuretics at baseline (Supporting Information S1: Table 3).

**Table 2:** Baseline characteristics of the intention‐to‐treat population.

	Apixaban, *N* = 11	Placebo, *N* = 14
Sex assigned at birth		
Male	2 (18.2%)	2 (14.3%)
Female	9 (81.8%)	12 (85.7%)
Age, years	64.1 (6.0)	63.6 (9.0)
Height, cm	161.8 (13.0)	162.3 (6.8)
Weight, kg	76.8 (61.0–94.0)	65.2 (55.0–69.8)
Body mass index, kg/m^2^	29.2 (26.1–32.2)	24.0 (21.8–25.1)
Ethnicity		
Caucasian	11 (100%)	13 (92.9%)
Aboriginal or Torres Strait Islander	0 (0%)	1 (7.1%)
Employment status		
Part‐time	0 (0.0%)	1 (7.1%)
Full‐time	2 (18.2%)	2 (14.3%)
Home‐duties	1 (9.1%)	1 (7.1%)
Disability preventing work	4 (36.4%)	3 (21.4%)
Retired	4 (36.4%)	6 (42.9%)
Unemployed	0 (0.0%)	1 (7.1%)
Highest level of education		
Secondary school	6 (54.5%)	6 (42.9%)
Trade school	3 (27.3%)	1 (7.1%)
Diploma	0 (0.0%)	4 (28.6%)
Degree	2 (18.2%)	2 (14.3%)
Drink alcohol	7 (63.6%)	9 (64.3%)
Ever smoked	3 (27.3%)	6 (42.9%)
Average daily cigarette quantity^a^	20.0 (2.0–30.0) *n* = 3	20.0 (15.0–20.0) *n* = 6
Years smoked^a^	29.0 (7.0–43.0) *n* = 3	24.5 (17.0–42.0) *n* = 6
Left ventricular ejection fraction %^a^	60 (55–65)	61.5 (58–70) *n* = 10
FEV1, percent predicted value	87.0 (75.0–103.0)	90.5 (75.0–104.0)
FVC, percent predicted value	85.0 (73.0–112.0)	103.0 (81.0–111.0)
FEV1/FVC ratio	77.0 (74.0–88.0)	74.5 (70.0–84.0)
Modified NYHA/WHO Functional Class I	0 (0.0%)	1 (7.1%)
Modified NYHA/WHO Functional Class II	3 (27.3%)	8 (57.1%)
Modified NYHA/WHO Functional Class III	8 (72.7%)	4 (28.6%)
Modified NYHA/WHO Functional Class IV	0 (0.0%)	1 (7.1%)
Six minute walk distance, meters	425 (332–492)	454 (350–522)
Borg dyspnea index	3 (3–4)	3 (2–3)
Resting mean pulmonary arterial pressure, mmHg	32.0 (26.0–42.0)	32.5 (28.0–36.0)
Resting pulmonary vascular resistance, Woods units^a^	3.9 (3.0–6.4)	3.5 (3.1–4.8) *n* = 13
Resting pulmonary arterial wedge pressure, mmHg	8.0 (5.0–10.0)	10.0 (7.0–14.0)
Left ventricular end diastolic pressure, mmHg^a^	16.0 (10.0–20.0) *n* = 6	18.0 (5.0–24.0) n = 3
Cardiac Index, liters/minute^a^	2.5 (2.1–2.6) *n* = 5	2.7 (2.1–4.3) *n* = 7
Duration since PAH diagnosis to baseline, years	0.52 (0.33–1.97)	1.35 (0.67–2.14)
Duration SSc onset to baseline, years	7.95 (2.18–23.63)	16.28 (7.01–30.56)
Diffuse scleroderma	3 (27.3%)	2 (14.3%)
Limited scleroderma	8 (72.7%)	12 (85.7%)

*Note:* Frequency presented as *n* (%) for categorical variables, mean (SD) for normally distributed continuous variables, and median (IQR) for non‐normally distributed continuous variables.

Abbreviations: FEV1 = forced expiratory volume in one second, FVC = forced vital capacity, N = total number of patients, n = number of patients, NYHA/WHO = New York Heart Association/World Health Organization, PAH = pulmonary arterial hypertension, RHC = right heart catheterization, SSc = systemic sclerosis.

a Data not available for all randomized participants.

### 3.2 Primary Outcome

A total of 28 suspected CWEs were presented to the endpoint adjudication committee following a median (± IQR) of 3.00 (2.57–3.01) years apixaban treatment versus 2.37 (1.90–2.99) years placebo (*p* = 0.056). All clinical worsening features that were adjudicated for the composite primary endpoint are reported in Table 3.

**Table 3:** Composite primary endpoint requisite events ever presenting for adjudication.

Primary endpoint criteria	Apixaban, *n* = 6	Placebo, *n* = 8
1. Death (all‐cause mortality)	1 (1)	2 (2)
2. Hospitalization for worsening of PAH meeting clinical criteria for need for lung transplantation	1 (1)	0 (0)
3. Initiation of parenteral (subcutaneous and intravenous) prostanoid therapy	1 (1)	2 (2)
4. Hospitalization meeting clinical criteria for initiation of chronic oxygen therapy	2 (2)	3 (3)
5. Reduction from baseline in 6MWD by 15%, confirmed by two consecutive 6MWTs completed on different days, ideally within 2 weeks of one another	6 (5)	9 (5)
6. An increase from baseline in modified NYHA/WHO functional class (except for participants already in Functional Class IV)	7 (5)	10 (6)
Appearance or worsening of signs or symptoms of right heart failure, that may include:		
7. Dyspnea at rest	2 (2)	3 (2)
8. Exertional dyspnea	3 (3)	5 (3)
9. Orthopnoea	0 (0)	0 (0)
10. Oxygen desaturation	2 (2)	0 (0)
11. Peripheral edema	5 (5)	3 (3)
12. Pulmonary edema	0 (0)	0 (0)
Need for additional PAH specific therapy that may include:		
13. Endothelin receptor antagonists	1 (1)	2 (2)
14. Inhaled prostanoids	0 (0)	1 (1)
15. Intravenous diuretics	2 (2)	5 (3)
16. PDE‐5 inhibitors	5 (4)	5 (5)
17. Other additional PAH specific therapy	1 (1)	1 (1)

*Note:* Numbers presented are total instances (number participants) of a requisite event adjudicated between baseline to 30 days following study drug cessation. Any singular instance of Items 1–4, or combinations of at least two features of disease progression Items 5–17, were adjudicated as clinical worsening events, where time to the first such event was analyzed as the primary endpoint, and all subsequent events were analyzed within the exploratory endpoints.

Abbreviations: 6MWD = distance walked in six minutes, 6MWT = six minute walk test, *n* = total number of participants presented for adjudicated of composite primary endpoint, NYHA/WHO = New York Heart Association/World Health Organization, PAH = pulmonary arterial hypertension, PDE‐5 = phosphodiesterase type‐5.

**Figure 3:**
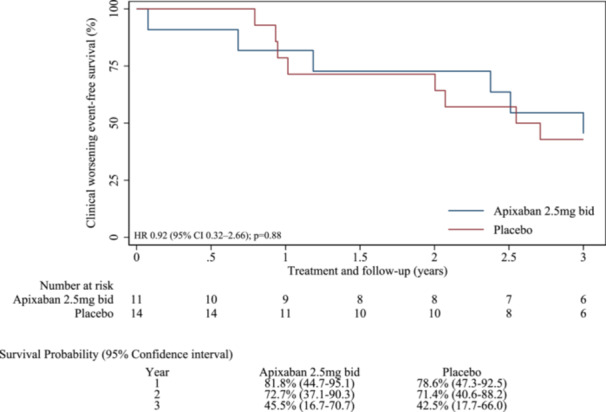
Kaplan–Meier survival curves for time from commencement of treatment to primary endpoint by treatment allocation.

The Kaplan–Meier survival curve and event‐free proportion estimates over time are displayed in Figure 3. Apixaban had no effect compared to placebo on TtCW from commencement until 30 days after discontinuation of study drug, and the Cox proportional hazard ratio of placebo:treatment was 0.92 (95% CI 0.32–2.66), *p* = 0.88.

### 3.3 Exploratory Endpoints

The total number of CWEs throughout the study period did not differ between treatment groups, with at least one CWE being experienced by 6 (54.5%) apixaban treated versus 8 (57.1%) placebo participants (Table 3). Among participants experiencing CWEs there was a median (±IQR) 3.5 (3.0–7.0) events per apixaban participant versus 5.0 (2.5–6.5) events per placebo participant. Of all‐cause deaths during the study, none contributed to the primary endpoint survival time because they were not the first CWE experienced by any participant. All‐cause mortality (adjusted for time since diagnosis of PAH) did not differ between treatment groups, with no deaths at 12 months and one in each treatment group at 24 months. By 36 months follow‐up, one participant in the apixaban group versus two controls was deceased. By the end of up to 3 years follow‐up, the proportion of participants on PAH combination therapy increased to 7 (63.6%) apixaban treated versus 10 (71.4%) placebo participants. The addition of intravenous prostanoid and increased use of diuretics coincided with reduced use of vasodilators overall (Supporting Information S1: Table 3). Whether for worsening of PAH, comorbidities or adverse events, the placebo group was observed to commence a greater total number of new medications per participant per year, median (±IQR) 2.0 (1.7–2.5) versus 1.3 (1.0–2.0) in apixaban treated participants (Supporting Information S1: Table 4). Nevertheless, there was no between‐group difference in uptake of any particular medication.

**Figure 4:**
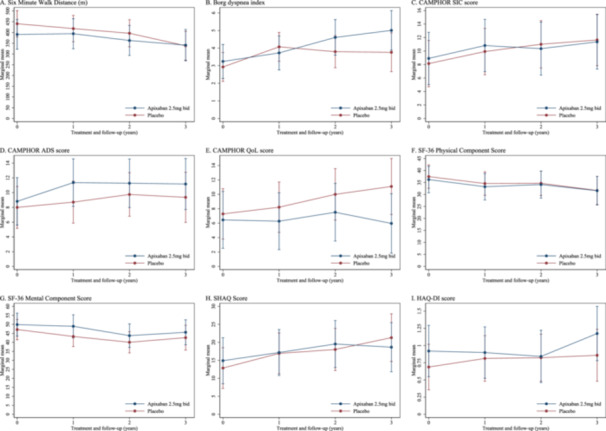
Multilevel mixed‐effects linear regression models of exploratory outcome measures. The marginal effect (95% confidence interval displayed by error bars) of the full interaction between treatment and time is displayed for each of the continuous exploratory outcomes: (A) Six minute walk distance in meters; (B) Borg dyspnea index on a scale from 0 *Nothing at all*, to 10 *Very, very severe* (maximal breathlessness); (C) PAH‐related symptomatic impairment up to a maximum of 25 points on the CAMPHOR; (D) CAMPHOR activity (disability) score, from a 15 item scale where 0 is able to do on their own without difficulty, 1 is able to do on their own with difficulty, and 2 is unable to do on own, such that worse disability reaches a maximum 30 points; (E) CAMPHOR overall quality of life scores negatively weighted to worsening, up to a maximum of 25 points; (F) Percentage of total possible SF‐36 physical component score;* (G) Percentage of total possible SF‐36 mental component scores;* (H) Total SHAQ Visual Analog Scale selections from 0 to 10, with 0 being *no functional limitation* and 10 being *severe functional limitation*, combined across six areas of SSc‐related limitation; (I) HAQ‐DI is the mean of 8 category scores from the 20‐item Sanford Health Assessment Questionnaire, a disability index where items are rated from 0 to 3, where higher scores indicate greatest disability. *SF‐36 component scores are averaged values from the physical and mental scales in the Medical Outcomes Study Short Form‐36, ranging from 0 to 100, where 0 is the presence of worst possible limitations and higher scores represent more favorable physical or mental health state.

Apart from participants already in Functional Class IV, a worsening in modified NYHA/WHO functional class between baseline and end of study treatment was observed in 5 (45.5%) apixaban treated and 6 (46.2%) placebo participants. There was no difference between treatment groups for annual change in 6MWD and corresponding Borg dyspnea index (Figure 4A,B, respectively). A high proportion of participants experienced overall decline in 6MWD between baseline and end of study treatment (9 (81.8%) apixaban vs. 10 (71.4%) placebo), although fewer participants reached the ≥ 15% decline indicative of disease progression (6 (54.5%) apixaban vs. 6 (42.9%) placebo) at any time during treatment. Only a modest increase in severity of median (±IQR) 1 (0.0–2.0) Borg dyspnea index points between baseline and end of study treatment was observed for both groups.

Group aggregate data for study assessments conducted over the duration of the study are provided within Supporting Information S1: Tables 5 and 6. There were no differences between the groups in any physical examination characteristics, vital signs, echocardiography results, or routine laboratory results. An increase in N‐terminal prohormone B‐type natriuretic peptide (NT‐proBNP) levels between baseline and 2 years follow‐up was observed in the majority of participants, 10 (90.9%) apixaban treated and 13 (92.9%) controls, with the placebo group experiencing a numerically larger increase from baseline to 2 years treatment (median (±IQR) 480.5 (58–747) ng/L increase in NT‐proBNP for placebo versus 86 (16–146) ng/L increase in NT‐proBNP for apixaban treated participants).

### 3.4 Patient Reported Outcome Measures

There were no between‐group differences in participant reported outcome measures at any time point (Figure 4C–I). Overall change from baseline to end of study treatment saw a cumulative burden over time in symptomatic impairment, physical disability, and perceived quality of life. Over the duration of the study, there was no between‐group difference in utilization of General Practitioner or Specialist services, nor in the number or duration of all‐cause hospitalizations (Supporting Information S1: Table 4). Although only very few patients experienced PAH‐related hospitalizations during the study, there was a tendency for greater median (±IQR) in‐patient length of stay of 34 (12–56) days per year per participant in the apixaban treatment group versus 7.7 (0.3–8.7) days per year per participant in the placebo group. The apixaban treatment group also demonstrated more PAH‐related hospitalizations per year per participant compared with placebo participants (Supporting Information S1: Table 4).

### 3.5 Safety Data

Treatment‐emergent adverse events occurring up to 7 days post‐study drug cessation are summarized in Table 4. There was no difference between treatment groups in the total number of AEs experienced per participant (median (±IQR) 15.0 (7.0–27.0) AEs per apixaban treated compared to 13.0 (7.0–19.0) AEs per placebo). Among 386 total adverse events, there was no between‐group difference in proportion of events that were serious adverse events (17/171 (9.9%) total AEs experienced by apixaban treated participants were SAEs, compared to 16/215 (7.4%) for placebo).

**Table 4:** Treatment‐emergent adverse events by treatment allocation.

	Apixaban, *N* = 11	Placebo, *N* = 14
Abnormal bleeding, all events	49 (54.5%)	63 (64.3%)
Epistaxis	31 (27.3%)	42 (42.9%)
Gastrointestinal abnormal bleeding	4 (27.3%)	3 (21.4%)
Hemorrhoids	1 (9.1%)	0 (0.0%)
Venepuncture site abnormal bleed	6 (27.3%)	0 (0.0%)
Other abnormal bleeding	7 (27.3%)	18 (42.9%)
Abnormal laboratory values	4 (18.2%)	3 (14.3%)
Iron deficiency anemia	12 (81.8%)	4 (28.6%)
Bruising	2 (9.1%)	8 (14.3%)
Cardiovascular disease, all events	3 (18.2%)	7 (28.6%)
Angina	2 (9.1%)	0 (0.0%)
Arrhythmia	1 (9.1%)	6 (21.4%)
Takotsubo cardiomyopathy	0 (0.0%)	1 (7.1%)
Cancer, all events	7 (18.2%)	4 (21.4%)
Basal cell carcinoma	1 (9.1%)	2 (14.3%)
Squamous cell carcinoma	6 (18.2%)	2 (14.3%)
Chest pain	2 (18.2%)	0 (0.0%)
Dyspnea	2 (18.2%)	3 (14.3%)
Fatigue	0 (0.0%)	1 (7.1%)
Gastrointestinal problems	6 (27.3%)	4 (21.4%)
Heart failure	0 (0.0%)	8 (21.4%)
Hypotension	2 (18.2%)	1 (7.1%)
Infection, all events	34 (90.9%)	50 (100%)
Dental infection	1 (9.1%)	1 (7.1%)
Ear infection	3 (18.2%)	1 (7.1%)
Eye infection	1 (9.1%)	1 (7.1%)
Gastrointestinal infection	2 (18.2%)	3 (21.4%)
Lower respiratory tract infection	5 (27.3%)	16 (64.3%)
Upper respiratory tract infection	11 (54.5%)	24 (92.9%)
Urinary tract infection	7 (36.4%)	0 (0.0%)
Skin infection	4 (36.4%)	3 (21.4%)
Viral infection	0 (0.0%)	1 (7.1%)
Mental health	6 (27.3%)	2 (14.3%)
Nasal congestion	2 (18.2%)	2 (14.3%)
Other	9 (63.6%)	20 (64.3%)
PAH	6 (36.4%)	5 (28.6%)
Pain	4 (27.3%)	4 (21.4%)
Peripheral edema	1 (9.1%)	5 (28.6%)
Rash	2 (18.2%)	2 (7.1%)
Serious adverse event	17 (72.7%)	16 (42.9%)
SSc‐related complication other than PAH	11 (72.7%)	14 (50.0%)
Thrombosis	0 (0.0%)	1 (7.1%)
Trauma	7 (36.4%)	5 (35.7%)

*Note:* Numbers presented are total treatment‐emergent adverse events up to 7 days after last study drug intake (percentage of patients in which they occurred).

Abbreviations: PAH = pulmonary arterial hypertension, SSc = systemic sclerosis.

The adverse event diagnoses occurring with greatest frequency out of 386 total events were 112 (29.0%) abnormal bleeding episodes, 84 (21.8%) infections, 25 (6.5%) complications of SSc other than PAH such as digital ulcers and 16 (4.1%) iron deficiency anemia (Table 4). Abnormal bleeding events were predominantly 73/112 (65.2%) epistaxis episodes, occurring at similar frequency between treatment groups in 3/11 (27.3%) apixaban treated compared to 6/14 (42.9%) placebo participants. Ongoing mild to moderate epistaxis necessitated premature withdrawal from the study of one apixaban treated and two placebo participants. Notably, three apixaban treated participants and no placebo patients reported abnormal bleeding from a venipuncture site and iron deficiency anemia requiring corrective therapy was reported in 9 (81.8%) apixaban treated participants compared to 4 (28.6%) placebo participants.

More than twice as many placebo (13 (92.9%) vs. 6 (54.5%) apixaban) treated participants experienced upper respiratory tract, or lower respiratory tract infections (9 (64.3%) placebo vs. 3 (27.3%) apixaban) between baseline and end of study. Conversely, 0 placebo and 4 (36.4%) apixaban treated participants reported urinary tract infection during the study (Table 4).

Anti‐factor Xa assays after at least 6 months study drug treatment confirmed bioavailability of apixaban in the treatment group (median (±IQR) 1.27 (0.82–1.50) IU/mL anti‐factor Xa activity) and absence of anti‐factor Xa activity in the placebo group (median (±IQR) 0.00 (0.00–0.01) IU/mL). The results in the apixaban group were not suggestive of nonadherence. Pill count at follow‐up visits found that all participants exceeded 95% adherence to the treatment regimen during the time that they remained in the study (median (±IQR) 98.6% (97.4–99.2) compliance to apixaban versus 97.6% (95.7–99.4) compliance to placebo). Excluding participants that prematurely withdrew from the study prior to completing 80% of the study duration (1 apixaban, 3 placebo), the per‐protocol Cox proportional hazard ratio 0.72 (95% CI 0.25–2.08), demonstrated no effect of apixaban compared to placebo on the TtCW. Nor did the sensitivity analysis using time since diagnosis of PAH by RHC as covariate to the TtCW, appreciably alter the intention to treat primary outcome (adjusted hazard ratio 0.86 (95% CI 0.6–1.18)).

## 4 Discussion

Although selection criteria in a complex disease meant recruitment was insufficiently powered to address the primary efficacy endpoint conclusively, the SPHInX study found no signal for benefit with anticoagulation as adjunct therapy in SSc‐PAH patients. There was no difference between treatment groups in event‐free survival, all‐cause mortality, or exploratory endpoints.

The SPHInX study remains the only randomized clinical trial to date to evaluate the efficacy of anticoagulation as additional therapy in patients with PAH. The SPHInX study was undertaken in response to calls from the scientific community for an RCT of anticoagulation in Group 1 PH patients, where the potential benefits of such treatment are still only supported by inconclusive observational research [15, 20, 44, 45, 46].

Our study highlights the challenges surrounding patient recruitment in the context of rare and complex disease. Individual study sites may treat too few eligible patients to invite to a study, necessitating wider collaboration and inclusion of numerous sites. In addition to increasing the cost of the RCT overall, each collaborator may have competing clinical and research interests that further impact feasibility of the collaboration, particularly in the context of an investigator‐initiated and led RCT, that is competitively funded. Many patients also declined to consent due to living too far away from a research center. When recruitment rates for SSc‐PAH patients slowed, we took the opportunity to amend the protocol to include iPAH, hPAH, and various CTD‐PAH patients. Unfortunately, the changes failed to improve timely participant uptake and the study remained considerably underpowered. With so few of these patients recruited, it was not possible to perform any analyses to detect whether subgroups could better tolerate anticoagulant therapy, so they were excluded from the intention to treat analysis.

There were 9 inclusion criteria to meet, and an extensive list of 24 exclusion criteria to ensure participant safety, which significantly hampered recruitment potential. Of particular relevance to recruitment for such a study, up to 6% of SSc patients exhibit intestinal telangiectasia or gastric antral vascular ectasiae (GAVE) which may bleed [20]. Moreover, unexplained iron deficiency anemia is a common feature of SSc [47]. Therefore, a history of gastrointestinal bleeding due to GAVE, or unexplained iron deficiency anemia in the last 12 months, were among the most important and commonly met exclusion criteria. With such a small sample, our results were likely confounded by between‐group differences in baseline characteristics, such as duration of SSc. Ultimately our sample size fell far short of the 85 per arm required to detect a two‐fold effect size if one was truly present.

Even though insufficiently powered to address the primary endpoint conclusively, the SPHInX study adds important new knowledge to this area of equipoise, indicating that any potential benefits of anticoagulation would likely be outweighed by the risks. Previous observational data in an Australian cohort (95% of whom had SSc‐PAH) demonstrated a substantial survival benefit with warfarin when administered in conjunction with advanced PAH therapy, even after adjusting for the severity of PAH and “combination” pulmonary vasodilator therapy [18]. However, that study did not report dose or duration of anticoagulation, nor did it systematically analyze safety data for signals regarding the extent of any harm that may have been caused by such treatment. The SPHInX RCT eliminated confounding by indication inherent to observational studies. Instead of warfarin, it utilized the more convenient oral anticoagulant apixaban which generally has more predictable bioavailability, fewer drug–drug interactions and does not require monitoring, being advantageous in this complex patient group in whom regular phlebotomy for International Normalized Ratio (INR) monitoring is impractical due to skin sclerosis [28]. The apixaban dose chosen was on balance of preclinical safety and efficacy data in other clinical settings where low‐dose 2.5 mg bid apixaban is therapeutically adequate for secondary prevention of thromboembolism, without the increased risk in bleeding observed at 5 mg bid full dose [28].

American cardiac and respiratory guidelines previously supported the use of anticoagulation in iPAH patients based only on observational evidence [33], and the current European guidelines remain in equipoise [48]. In contrast, use of anticoagulation is not currently considered “standard of care” in SSc‐PAH, based only on level “C” expert opinion [46, 49]. Under double‐blind, randomized, placebo‐controlled conditions, our SPHInX results could raise the level of evidence to “B” for Class III recommendation against the use of anticoagulation in SSc‐PAH.

Whilst a larger multinational RCT of oral anticoagulation in Group 1 PH may overcome the limitations of this study in future, such an undertaking poses even greater challenges than our national RCT. The greater financial cost and complexity in adherence to cross‐border research ethics requirements may meet with too many competing interests to justify for a rare disease. Our study was limited to SSc‐PAH and our interpretation is not generalizable to other Group 1 PH patient groups. Notwithstanding the inadequate sample size for detecting conclusive safety signals, the high frequency of treatment emergent iron deficiency anemia in this study suggests that even at low doses, risk may outweigh any benefit with anticoagulation in SSc‐PAH.

## Author Contributions

Mandana Nikpour directly accessed and verified the underlying data reported in the manuscript. Alicia Calderone was involved in project administration, data curation, and writing this manuscript. Dylan Hansen was responsible for the formal analysis and interpretation of study data. Wendy Stevens, Mandana Nikpour, Susanna M. Proudman, Eli Gabbay, Nathan Dwyer, Gregory Keir, Vivek Thakkar, Peter K. K. Wong, Anne Keogh, Andrew Burns, David S. Celermajer, and Joanne Sahhar conducted patient screening, study investigations, and supervision of data collection. Mandana Nikpour, Rachelle Buchbinder, David Prior, Harshal Nandurkar, Eli Gabbay, Susanna M. Proudman, Wendy Stevens, David S. Celermajer, Peter K. K. Wong, and Vivek Thakkar made substantial contributions to the manuscript from conceptualization of methodology and study design to funding acquisition. All authors had full access to all the data in the study, contributed to writing, editing, or reviewing drafts of this work critically for important intellectual content, and accept responsibility for the final version to be published.

## Ethics Statement

Institutional Review Board approval was granted by the Human Research Ethics Committees of St. Vincent's Hospital Melbourne, Royal Perth Hospital, University of Western Australia, and Menzies Research Institute of Tasmania. All procedures followed were in accordance with institutional guidelines, acknowledged by the Governance offices of all hospitals involved in the trial (Fiona Stanley Hospital, Gold Coast University Hospital, Liverpool Hospital, Monash Health, Princess Alexandra Hospital, Royal Adelaide Hospital, Royal Hobart Hospital, Royal Prince Alfred Hospital, St Vincent's Hospital Sydney, The Alfred Hospital, and The Queen Elizabeth Hospital).

## Consent

Patients provided written informed consent prior to study enrollment and treatment allocation, following adequate explanation of the aims, methods, objectives, and potential hazards of the trial by the responsible investigator.

## Conflicts of Interest

M.N. holds an NHMRC Investigator Grant (GNT1176538). M.N. has received research support grants from Janssen and Boehringer Ingelheim. M.N. has received honoraria or consultancies from Janssen, AstraZeneca, GSK, and Boehringer Ingelheim. S.M.P. has received honoraria from Janssen and Boehringer Ingelheim. N.D. has received research support grants from Bayer, GSK, Johnson & Johnson, MSD, and United Therapeutics. G.K. has received a research support grant from Janssen and consultancies from Actelion, Boehringer Ingelheim, Menarini, and Roche. P.K.K.W. has received research support from AbbVie, Janssen, Novartis, Pfizer, and UCB. P.K.K.W. has received consultancies from AbbVie, Janssen, Lilly, Novartis, and Pfizer. A.K. has received consultancies from Pfizer. D.P. has received consultancies from Janssen, Novartis, and Boehringer Ingelheim. H.N. has received consultancies from BMS‐Pfizer and Boehringer Ingelheim. J.S. has received honoraria and research support from Janssen and Boehringer Ingelheim. W.S. has received consultancies from Janssen, Boehringer Ingelheim, Certa Therapeutics, GSK, and Merck. R.B. is supported by an NHMRC Investigator Grant (APP1194483). The authors A.C., D.H., E.G., G.K., V.T., A.B., and D.S.C. declare that there is no conflict of interest.

## Supporting information

Supporting File 1

Supporting File 2

## Data Availability

Data sharing is outside the scope of the Human Research Ethics Committee approvals and written Participant Information Sheet and Consent Forms signed for the conduct of this study. The data that support the findings of this study are included within the article or uploaded as Supporting Files.
