# Supplementary material for: A Multicenter, Double‐Blind, Placebo‐Controlled, Randomized Clinical Trial of Oral Anticoagulation in Systemic Sclerosis‐Related Pulmonary Arterial Hypertension—Results From the SPHInX Study
Source: Pulm Circ. 2026 Jun 8;16(2):e70331. doi: 10.1002/pul2.70331 (PMC13244262; doi:10.1002/pul2.70331)
Supplement: Supplementary file 1 — Supporting File 1 [file PUL2-16-e70331-s001.docx]

**Supplementary Table 1. Underlying systemic sclerosis features and autoantibody profile**

|  | **Apixaban, N=11** | **Placebo, N=14** |
| --- | --- | --- |
| Skin thickening of the fingers of both hands extending proximal to MCP joint | 4 (36.4%) | 7 (50.0%) |
| Puffy fingers | 3 (27.3%) | 4 (28.6%) |
| Sclerodactyly distal to the MCP joints but proximal to the PIP joints | 7 (63.3%) | 10 (73.3%) |
| Digital tip ulcers | 1 (9.1%) | 5 (35.7%) |
| Fingertip pitting scars | 6 (54.5%) | 9 (64.3%) |
| Telangiectasia | 10 (90.9%) | 14 (100%) |
| Abnormal nailfold capillaries | 6 (54.5%) | 11 (78.6%) |
| Pulmonary Arterial Hypertension | 11 (100%) | 14 (100%) |
| Interstitial lung disease | 2 (18.2%) | 2 (14.3%) |
| Raynaud’s phenomenon | 11 (100%) | 14 (100%) |
| SSc-related autoantibodies | 8 (72.7%) | 11 (78.6%) |
| Total score on ACR/EULAR Criteria for Classification of SSc | 17.0 (14-19) | 19.5 (19-24) |
| Duration SSc since first non-RP symptom (years) | 8.95 (2.18-21.63) | 13.34 (4.20-25.12) |
| Calcinosis as first non-RP symptom | 1 (9.1%) | 1 (7.1%) |
| Digital ulcer as first non-RP symptom | 2 (18.2%) | 1 (7.1%) |
| Pulmonary Arterial Hypertension as first non-RP symptom | 1 (9.1%) | 1 (7.1%) |
| Severe reflux as first non-RP symptom | 2 (18.2%) | 2 (14.3%) |
| Skin tightening as first non-RP symptom | 2 (18.2%) | 3 (21.4%) |
| Swollen fingers as first non-RP symptom | 3 (27.3%) | 5 (35.7%) |
| Telangiectasia as first non-RP symptom | 0 (0.0%) | 1 (7.1%) |
| ANA positive | 11 (100%) | 14 (100%) |
| ANA Centromere pattern | 7 (63.3%) | 9 (64.3%) |
| ANA Homogenous pattern | 2 (18.2%) | 1 (7.1%) |
| ANA Nucleolar pattern | 3 (27.3%) | 4 (28.6%) |
| ANA Speckled pattern | 2 (18.2%) | 3 (21.4%) |
| ENA positive | 3 (27.3%) | 3 (21.4%) |
| anti-dsDNA antibody positive | 0 (0.0%) | 0 (0.0%) |
| anti-RNA polymerase III antibody positive | 2 (18.2%) | 1 (7.1%) |
| anti-phospholipid antibody positive | 1 (9.1%) | 0 (0.0%) |
| anti-beta2 glycoprotein antibody positive | 0 (0.0%) | 1 (7.7%) |
| Lupus anticoagulant positive | 0 (0.0%) | 0 (0.0%) |
| Rheumatoid Factor positive | 3 (27.3%) | 5 (33.3%) |
| anti-CCP antibody positive | 0 (0.0%) | 0 (0.0%) |

Frequency data presented as n (%) for categorical variables, and median (interquartile range) for non-normally distributed continuous variables.

*Abbreviations:* ACR/EULAR = American College of Rheumatology/ European League Against Rheumatism; ANA = antinuclear antibody; CCP = cyclic citrullinated peptides; dsDNA = double stranded DNA; ENA = extractable nuclear antigens; MCP = metacarpophalangeal; PIP = proximal interphalangeal; RP = Reynaud’s Phenomenon; SSc = Systemic Sclerosis.

**Supplementary Table 2. Baseline clinical co-morbidities**

|  | **Apixaban, N=11** | **Placebo, N=14** |
| --- | --- | --- |
| Reflux Oesophagitis | 10 (90.1%) | 14 (100.0%) |
| Oesophageal Stricture | 2 (18.2%) | 1 (7.1%) |
| Oesophageal Dysmotility | 4 (36.4%) | 5 (35.7%) |
| Bowel Dysmotility or Pseudo-obstruction | 0 (0.0%) | 4 (28.6%) |
| Pulmonary Fibrosis or Interstitial Lung Disease | 2 (18.2%) | 4 (28.6%) |
| Diabetes | 1 (9.1%) | 0 (0.0%) |
| Systemic Hypertension | 7 (63.6%) | 5 (35.7%) |
| Hypercholesterolaemia | 3 (27.3%) | 7 (50.0%) |
| Cardiac involvement  Angina  Myocardial Infarction  Prosthetic Valve  Left ventricular dysfunction  Diastolic Dysfunction  Conduction defect  Arrhythmia | 3 (27.3%)  0 (0.0%)  0 (0.0%)  0 (0.0%)  0 (0.0%)  0 (0.0%)  2 (18.2%)  1 (9.1%) | 4 (28.6%)  0 (0.0%)  1 (7.1%)  0 (0.0%)  0 (0.0%)  1 (7.1%)  0 (0.0%)  2 (14.3%) |
| Pericardial effusion | 1 (9.1%) | 2 (14.3%) |
| Pulmonary embolus | 0 (0.0%) | 0 (0.0%) |
| Stroke or Transient Ischaemic Attack | 0 (0.0%) | 1 (7.1%) |
| Deep vein thrombosis | 0 (0.0%) | 1 (7.1%) |
| Emphysema | 0 (0.0%) | 2 (14.3%) |
| Asthma | 0 (0.0%) | 1 (7.1%) |
| Myositis | 1 (9.1%) | 0 (0.0%) |
| Synovitis | 1 (9.1%) | 2 (14.3%) |
| Inflammatory arthritis | 0 (0.0%) | 2 (14.3%) |
| Digital ulcers | 7 (63.6%) | 8 (57.1%) |
| Digital gangrene/amputation | 1 (9.1%) | 1 (7.1%) |
| Cancer, non-terminal/in remission | 3 (27.3%) | 4 (28.6%) |
| Organ transplant | 0 (0.0%) | 0 (0.0%) |
| Iron deficiency anaemia of unknown origin in past 12 months | 0 (0.0%) | 0 (0.0%) |
| Gastric antral vascular ectasia, non-bleeding in past 12 months | 1 (9.1%) | 0 (0.0%) |
| Number of co-morbidities listed per patient | 7 (6-8) | 7 (4-10) |
| Number of previous surgical procedures listed per patient | 3 (2-5) | 2 (0-3) |

Frequency presented as n (%) for categorical variables, and median (interquartile range) for non-normally distributed continuous variables.

# **Supplementary Table 3. Baseline concomitant medication usage and changes to medication during the study**

|  | **Taking at baseline** | | **Ceased/Withheld** | | **Temporary use*** | | **New/Ongoing**† | |
| --- | --- | --- | --- | --- | --- | --- | --- | --- |
| **Medication category** | **Apixaban (N=11)** | **Placebo (N=14)** | **Apixaban (N=11)** | **Placebo (N=14)** | **Apixaban (N=11)** | **Placebo (N=14)** | **Apixaban (N=11)** | **Placebo (N=14)** |
| Endothelin Antagonists | 11 (100%) | 14 (100%) | 1 (9.1%) | 3 (21.4%) | - | - | 11 (100%) | 14 (100%) |
| Ambrisentan | 1 (9.1%) | 2 (14.3%) | - | - | - | - | 1 (9.1%) | 3 (21.4%) |
| Bosentan | 3 (27.3%) | 4 (28.6%) | - | 3 (21.4%) | - | - | 4 (36.4%) | 1 (7.1%) |
| Macitenan | 7 (63.6%) | 8 (57.1%) | 1 (9.1%) | - | - | - | 6 (54.5%) | 10 (71.4%) |
| PDE-5 Inhibitors | 3 (27.3%) | 8 (57.1%) | 0 (0.0%) | 1 (7.1%) | - | - | 7 (63.6%) | 10 (71.4%) |
| Sildenafil | 2 (18.2%) | 6 (42.9%) | - | 1 (7.1%) |  |  | 5 (45.5%) | 5 (35.7%) |
| Tadalafil | 1 (9.1%) | 2 (14.3%) | - | - |  |  | 2 (18.2%) | 5 (35.7%) |
| Prostanoid | - | - | - | - | 1 (9.1%) | 2 (14.3%) | 1 (9.1%) | 2 (14.3%) |
| Anticoagulant | - | - | - | - | 3 (27.3%) | 3 (21.4%) | 0 (0.0%) | 1 (7.1%) |
| Antiplatelet | 3 (27.3%) | 4 (28.6%) | 1 (9.1%) | 1 (7.1%) | - | - | 4 (36.4%) | 3 (21.4%) |
| Anti-hypertensive vasodilators | 6 (54.5%) | 8 (57.1%) | 4 (36.4%) | 3 (21.4%) | - | - | 3 (27.3%) | 5 (35.7%) |
| Diuretic | 5 (45.5%) | 7 (50.0%) | 2 (18.2%) | 0 (0.0%) | 2 (18.2%) | 3 (21.4%) | 6 (63.6%) | 9 (64.3%) |
| Anaesthetic | - | - | - | - | 2 (18.2%) | 0 (0.0%) | 0 (0.0%) | 2 (14.3%) |
| Analgesic | 1 (9.1%) | 3 (21.4%) | - | - | 4 (36.4%) | 3 (21.4%) | 3 (27.3%) | 5 (35.7%) |
| Antacid | 8 (72.7%) | 14 (100%) | 1 (9.1%) | 1 (7.1%) | 1 (9.1%) | 1 (7.1%) | 9 (81.8%) | 13 (92.9%) |
| Antibiotic | 0 (0.0%) | 1 (7.1%) | 0 (0.0%) | 1 (7.1%) | 11 (100%) | 14 (100%) | - | - |
| Antihistamine | 0 (0.0%) | 4 (28.6%) | - | - | 2 (18.2%) | 3 (21.4%) | 1 (9.1%) | 6 (42.9%) |
| Antimalarial | 1 (9.1%) | 1 (7.1%) | - | - | - | - | 1 (9.1%) | 2 (14.3%) |
| Antiviral | 0 (0.0%) | 1 (7.1%) | - | - | 1 (9.1%) | 3 (21.4%) | 0 (0.0%) | 1 (7.1%) |
| Immunosuppressant | 2 (18.2%) | 1 (7.1%) | 0 (0.0%) | 1 (7.1%) | 0 (0.0%) | 2 (14.3%) | 3 (27.3%) | 0 (0.0%) |
| Immunization | - | - | - | - | 5 (45.5%) | 6 (42.9%) | - | - |
| NSAID | - | - | - | - | 1 (9.1%) | 2 (14.3%) | - | - |
| Psychotropic | 2 (18.2%) | 6 (42.9%) | 0 (0.0%) | 2 (14.3%) | 0 (0.0%) | 4 (28.6%) | 5 (45.5%) | 7 (50.0%) |
| Statin | 2 (18.2%) | 6 (42.9%) | 0 (0.0%) | 1 (7.1%) | - | - | 3 (27.3%) | 5 (35.7%) |
| Steroid | 3 (27.3%) | 2 (14.3%) | 0 (0.0%) | 1 (7.1%) | 5 (45.5%) | 7 (50.0%) | 4 (36.4%) | 6 (42.9%) |
| Vitamins or Supplement | 6 (54.5%) | 9 (64.3%) | 1 (9.1%) | 0 (0.0%) | 9 (81.8%) | 5 (35.7%) | 9 (81.8%) | 13 (92.9%) |
| Iron supplement | 1 (9.1%) | 1 (7.1%) | - | - | 7 (63.6%) | 4 (28.6%) | 5 (45.5%) | 4 (28.6%) |
| Oxygen supplement | 1 (9.1%) | 0 (0.0%) | - | - | - | - | 3 (27.3%) | 3 (21.4%) |
| Uncategorized others | 5 (45.5%) | 7 (50.0%) | 1 (9.1%) | 1 (7.1%) | 3 (27.3%) | 8 (57.1%) | 6 (54.5%) | 10 (71.4%) |
| Total medications recorded | 78 | 131 | 11 | 16 | 131 | 166 | 113 | 191 |
| Total concurrent medications per participant | 7.0 (5.0-9.0) n=11 | 9.5 (6.0-12.0) n=14 | 1.0 (1.0-2.0) n=6 | 1.5 (1.0-3.0) n=8 | 9.0 (7.0-14.0) n=11 | 9.5 (6.0-17.0) n=14 | 5.0 (3.0-7.0) n=11 | 7.0 (5.0-11.0) n=14 |
| p-value of concurrent medications per participant | 0.169 | | 0.472 | | 0.956 | | 0.078 | |

Concomitant medication use is presented as n (%) of participants ever taking a given category of medication. Total concurrent medications are presented as the median (IQR) of concurrent medications used per participant across all categories. *Temporary includes withheld medications that were resumed and subsequently ceased.

†Ongoing includes withheld medications that were resumed and medications that had dosage altered during study, with no end date by the end of study.

**Supplementary Table 4. Health service utilization on a yearly basis**

|  | **Apixaban, N=11** | **Placebo, N=14** | ***p-*value** |
| --- | --- | --- | --- |
| Total all-cause hospitalisations per year* | 8.1 (n=9) | 11.2 (n=9) | . |
| All-cause hospitalisations per year per participant | 0.7 (0.3-1.0) | 1.0 (0.7-1.7) | 0.38 |
| Total length of all-cause in-patient stay per year, in days | 37.1 | 34.4 | . |
| Length of all-cause in-patient stay per year per participant, in days | 1.7 (1.0-6.0) | 1.0 (0.7-3.7) | 0.72 |
| Total PAH-related hospitalisations per year* | 2.9 (n=2) | 4.2 (n=5) | . |
| PAH-related hospitalisations per year per participant | 1.4 (0.9-1.9) | 0.5 (0.3-1.4) | 0.25 |
| Length of PAH-related in-patient stay per year, in days | 67.9 | 25.6 | . |
| Length of PAH-related in-patient stay per year per participant, in days | 34.0 (12.0-56.0) | 7.7 (0.3-8.7) | 0.053 |
| Total General Practitioner visits per year* | 30.9 (n=9) | 45.1 (n=13) | . |
| General Practitioner visits per year per participant | 1.7 (1.3-5.5) | 1.6 (1.3-5.3) | 0.92 |
| Total Specialist visits per year* | 31.5 (n=9) | 60.6 (n=12) | . |
| Total Specialist visits per year per participant | 2.3 (0.7-7.0) | 2.9 (1.6-6.3) | 0.62 |
| Total new medications commenced per year | 22.8 (n=11) | 26.1 (n=12) | . |
| New medications commenced per year per participant | 1.3 (1.0-2.0) | 2.0 (1.7-2.5) | 0.049 |

Unless otherwise stated, frequency presented as median (interquartile range), with a *p*-value calculated by Wilcoxon rank-sum test.

*Data not available for all randomised participants; n=number of participants with data included.

# **Supplementary Table 5. Physical examinations and vital signs from baseline to end of study participation**

|  | **Apixaban, N=11** | **Placebo, N=14** | ***p-*value** |
| --- | --- | --- | --- |
| Change in total body weight, kg | -3.7 (-5.8 – 0.7) | -2.6 (-5.4 – -0.4) | 0.93 |
| 12-lead ECG Sinus Rhythm at all times, n (%) | 10 (90.9%) | 13 (92.9%) | 0.86 |
| Atrial Fibrillation, ever during study, n (%) | 1 (9.1%) | 1 (7.1%) | 0.86 |
| Left ventricular Ejection Fraction <45%, ever during study, n (%) | 0 (0%) | 0 (0%) | - |
| Change in left ventricular ejection fraction % * | 1 (-5 – 8) | 1 (-15 – 5), n=10 | 0.31 |
| Lung crackles, ever during study, n (%) | 5 (45.5%) | 5 (35.7%) | 0.62 |
| Raised Jugular Venous Pressure, ever during study, n (%) | 6 (54.5%) | 5 (35.7%) | 0.35 |
| Peripheral oedema, ever during study, n (%) | 8 (72.7%) | 6 (42.9%) | 0.14 |
| Change in supine heart rate, beats per minute | 1 (-5 – 5) | 0.5 (-15 – 5) | 0.78 |
| Change in supine systolic blood pressure, mmHg | -19 (-23 – 10) | -5 (-10 – 13) | 0.06 |
| Change in supine diastolic blood pressure, mmHg | -9 (-16 – 5) | 1 (-2 – 10) | 0.14 |
| Change in standing heart rate, beats per minute * | -2 (-9 – 13) | -1.5 (-9.5 – 10.5), n=12 | 0.95 |
| Change in standing systolic blood pressure standing, mmHg * | -7 (-12 – 1) | -5 (-5 – 2), n=11 | 0.53 |
| Change in standing diastolic blood pressure standing, mmHg * | -8 (-12 – 5) | 0 (-4 – 4), n=11 | 0.72 |

Data presented as n (%) for categorical variables, and median (IQR) for non-normally distributed continuous variables.

*Data not available for all randomised participants; n=number of participants with data included.

# **Supplementary Table 6. Routine laboratory results over the duration of the study including frequency outside of reference range**

| **Full blood test constituent** | **Reference range** | **Units** | **Apixaban N=11** | **Placebo N=14** | **p** |
| --- | --- | --- | --- | --- | --- |
| Haemoglobin at Baseline | Male 128-175, Female 113-159 | g/L | 129 (118-133) | 122.5 (113-129) | 0.44 |
| *Frequency below range* |  |  | *1 (9.1%)* | *4 (28.6%)* | *0.23* |
| Haemoglobin at 1 year | Male 128-175, Female 113-159 | g/L | 121 (113-126) | 119 (114-145) | 0.70 |
| *Frequency below range* |  |  | *3 (27.3%)* | *4 (28.6%)* | *0.94* |
| Haemoglobin at 2 years* | Male 128-175, Female 113-159 | g/L | 122 (114-143), n=10 | 126 (121-139.5), n=12 | 0.34 |
| *Frequency below range* |  |  | *2 (20.0%)* | *2 (16.7%)* | *0.84* |
| Haemoglobin at 3 years* | Male 128-175, Female 113-159 | g/L | 123 (114-130), n=9 | 121 (103-134), n=7 | 0.87 |
| *Frequency below range* |  |  | *1 (11.1%)* | *3 (42.9%)* | *0.15* |
| White Cell Count at Baseline | 3.9-12.7 | x10^9^/L | 7.3 (6.1-7.8) | 7.0 (5.8-9.0) | 0.89 |
| White Cell Count at 1 year | 3.9-12.7 | x10^9^/L | 6.1 (5.1-7.4) | 6.6 (5.5-7.5) | 0.58 |
| White Cell Count at 2 years* | 3.9-12.7 | x10^9^/L | 6.1 (5.7-6.6), n=10 | 6.1 (5.1-7.9), n=12 | 0.95 |
| *Frequency below range* |  |  | *0 (0.0%)* | *1 (8.3%)* | *0.35* |
| White Cell Count at 3 years* | 3.9-12.7 | x10^9^/L | 6.3 (5.2-7.9), n=9 | 6.3 (5.0-6.9), n=7 | 0.96 |
| Red Blood Cells at Baseline* | Male 4.0-5.8, Female 3.6-5.3 | x10^12^/L | 4.4 (4.0-4.5), n=10 | 4.5 (4.1-5.0), n=13 | 0.39 |
| *Frequency below range* |  |  | *1 (10.0%)* | *0 (0.0%)* | *0.24* |
| Red Blood Cells at 1 year* | Male 4.0-5.8, Female 3.6-5.3 | x10^12^/L | 4.1 (3.9-4.3), n=10 | 4.5 (4.1-4.8), n=13 | 0.044 |
| *Frequency below range* |  |  | *2 (20.0%)* | *0 (0.0%)* | *0.092* |
| Red Blood Cells at 2 years* | Male 4.0-5.8, Female 3.6-5.3 | x10^12^/L | 4.4 (4.0-4.7), n=9 | 4.6 (4.3-5.0), n=11 | 0.14 |
| *Frequency below range* |  |  | *1 (11.1%)* | *0 (0.0%)* | *0.26* |
| Red Blood Cells at 3 years* | Male 4.0-5.8, Female 3.6-5.3 | x10^12^/L | 4.3 (4.1-4.5), n=9 | 4.4 (4.2-4.9), n=6 | 0.52 |
| *Frequency below range* |  |  | *0 (0.0%)* | *1 (16.7%)* | *0.21* |
| Platelets at Baseline | 150-396 | x10^9^/L | 223 (164-240) | 243 (193-285) | 0.25 |
| *Frequency below range* |  |  | *0 (0.0%)* | *2 (14.3%)* | *0.19* |
| Platelets at 1 year | 150-396 | x10^9^/L | 199 (171-253) | 207.5 (193-296) | 0.38 |
| *Frequency below range* |  |  | *0 (0.0%)* | *2 (14.3%)* | *0.19* |
| Platelets at 2 years* | 150-396 | x10^9^/L | 212.5 (183-253), n=10 | 211.5 (159.5-293.5), n=12 | 0.92 |
| *Frequency below range* |  |  | *1 (10.0%)* | *3 (25%)* | *0.36* |
| Platelets at 3 years* | 150-396 | x10^9^/L | 233 (192-243), n=9 | 213 (180-275), n=7 | 0.96 |
| *Frequency below range* |  |  | *0 (0.0%)* | *1 (14.3%)* | *0.24* |
| Haematocrit at Baseline | Male 0.36-0.50, Female 0.32-0.42 | L/L | 0.40 (0.36-0.41) | 0.38 (0.35-0.40) | 0.39 |
| *Frequency below range* |  |  | *1 (9.1%)* | *0 (0.0%)* | *0.25* |
| Haematocrit at 1 year | Male 0.36-0.50, Female 0.32-0.42 | L/L | 0.37 (0.36-0.40) | 0.38 (0.35-0.42) | 0.83 |
| Haematocrit at 2 years* | Male 0.36-0.50, Female 0.32-0.42 | L/L | 0.39 (0.37-0.43), n=10 | 0.39 (0.38-0.42), n=12 | 0.64 |
| *Frequency below range* |  |  | *0 (0.0%)* | *1 (8.3%)* | *0.35* |
| Haematocrit at 3 years* | Male 0.36-0.50, Female 0.32-0.42 | L/L | 0.39 (0.37-0.40), n=9 | 0.38 (0.33-0.43) n=7 | 0.71 |
| *Frequency below range* |  |  | *0 (0.0%)* | *1 (14.3%)* | *0.24* |
| Bilirubin at Baseline | Male <23, Female <14 | µmol/L | 8.0 (7.0-10.0) | 6.5 (5.0-8.0) | 0.14 |
| Bilirubin at 1 year* | Male <23, Female <14 | µmol/L | 7.5 (6.0-9.0), n=10 | 7.5 (5.0-15.0) | 0.91 |
| Bilirubin at 2 years* | Male <23, Female <14 | µmol/L | 7.0 (6.0-9.0), n=10 | 8.0 (5.1-14.5) n=12 | 0.57 |
| Bilirubin at 3 years* | Male <23, Female <14 | µmol/L | 7.0 (6.0-8.0), n=9 | 6.0 (4.0-11.0) n=7 | 0.67 |
| ALP at Baseline | Male 42-125, Female 36-106 | U/L | 85.0 (79-95) | 78.5 (68-98) | 0.58 |
| *Frequency above range* |  |  | *1 (9.1%)* | *2 (14.3%)* | 0.69 |
| ALP at 1 year | Male 42-125, Female 36-106 | U/L | 73.0 (69-88) | 79.5 (56-92) | 1.00 |
| *Frequency above range* |  |  | *0 (0.0%)* | *2 (14.3%)* | 0.19 |
| ALP at 2 years* | Male 42-125, Female 36-106 | U/L | 80.5 (73-96), n=10 | 79.5 (55-97) n=12 | 0.55 |
| *Frequency above range* |  |  | *2 (20.0%)* | *2 (16.7%)* | 0.84 |
| ALP at 3 years* | Male 42-125, Female 36-106 | U/L | 80.0 (71-81), n=9 | 70.0 (46-106) n=7 | 0.53 |
| *Frequency above range* |  |  | *1 (11.1%)* | *0 (0.0%)* | 0.36 |
| ALT at Baseline | Male 12-52, Female 9-36 | U/L | 15.0 (10-21) | 19.0 (13-22) | 0.25 |
| ALT at 1 year | Male 12-52, Female 9-36 | U/L | 12.0 (9-22) | 21.0 (16-25) | 0.04 |
| ALT at 2 years* | Male 12-52, Female 9-36 | U/L | 10.5 (9-16), n=10 | 18.5 (14.5-26.5), n=12 | 0.05 |
| ALT at 3 years* | Male 12-52, Female 9-36 | U/L | 14.0 (12-19), n=9 | 16.0 (14-23), n=7 | 0.34 |
| GGT at Baseline | Male <62, Female <38 | U/L | 20.0 (15-38) | 22.5 (15-44) | 0.62 |
| *Frequency above range* |  |  | *1 (9.1%)* | *5 (35.7%)* | *0.12* |
| GGT at 1 year | Male <62, Female <38 | U/L | 21.0 (11-33) | 25.0 (15-54) | 0.14 |
| *Frequency above range* |  |  | *1 (9.1%)* | *5 (35.7%)* | *0.12* |
| GGT at 2 years* | Male <62, Female <38 | U/L | 20.5 (11-34), n=10 | 29.5 (17.5-59.5), n=12 | 0.18 |
| *Frequency above range* |  |  | *1 (10.0%)* | *4 (33.3%)* | *0.19* |
| GGT at 3 years* | Male <62, Female <38 | U/L | 23.0 (10-30), n=9 | 36.0 (18-58), n=7 | 0.11 |
| *Frequency above range* |  |  | *1 (11.1%)* | *2 (28.6%)* | *0.38* |
| Albumin at Baseline | 33-46 | g/L | 39.0 (37-43) | 38.5 (37-40) | 0.51 |
| *Frequency above range* |  |  | *1 (9.1%)* | *1 (7.1%)* | *0.86* |
| Albumin at 1 year | 33-46 | g/L | 38.0 (34-41) | 40.0 (36-43) | 0.41 |
| *Frequency above range* |  |  | *1 (9.1%)* | *0 (0.0%)* | *0.14* |
| *Frequency below range* |  |  | *2 (18.2%)* | *0 (0.0%)* | *0.14* |
| Albumin at 2 years* | 33-46 | g/L | 38.5 (37-40), n=10 | 38.5 (36.5-43), n=12 | 0.64 |
| *Frequency below range* |  |  | *0 (0.0%)* | *1 (8.3%)* | *0.35* |
| Albumin at 3 years* | 33-46 | g/L | 38.0 (36-40), n=9 | 35.0 (34-39), n=7 | 0.18 |
| *Frequency below range* |  |  | *0 (0.0%)* | *1 (14.3%)* | *0.38* |
| Protein at Baseline | 60-80 | g/L | 76.0 (68-77) | 71.0 (64-75) | 0.28 |
| *Frequency above range* |  |  | *0 (0.0%)* | *1 (7.1%)* | *0.34* |
| Protein at 1 year | 60-80 | g/L | 71.0 (65-80) | 71.0 (65-76) | 1.00 |
| *Frequency above range* |  |  | *2 (18.2%)* | *0 (0.0%)* | *0.10* |
| Protein at 2 years* | 60-80 | g/L | 70.0 (67-73), n=10 | 72.0 (64.5-79), n=12 | 0.64 |
| *Frequency above range* |  |  | *0 (0.0%)* | *1 (8.3%)* | *0.35* |
| Protein at 3 years* | 60-80 | g/L | 71.0 (66-73), n=9 | 71.0 (64-75), n=7 | 0.92 |
| Sodium at Baseline | 135-143 | mmol/L | 140.0 (138-142) | 140.0 (139-141) | 0.56 |
| *Frequency above range* |  |  | *0 (0.0%)* | *1 (7.1%)* | *0.43* |
| *Frequency below range* |  |  | *0 (0.0%)* | *1 (7.1%)* | *0.43* |
| Sodium at 1 year | 135-143 | mmol/L | 140.0 (139-142) | 139.5 (137-141) | 0.40 |
| *Frequency above range* |  |  | *0 (0.0%)* | *1 (7.1%)* | *0.37* |
| Sodium at 2 years* | 135-143 | mmol/L | 142.0 (140-143), n=10 | 139.5 (138-141.5), n=12 | 0.067 |
| *Frequency above range* |  |  | *2 (20.0%)* | *0 (0.0%)* | *0.10* |
| Sodium at 3 years* | 135-143 | mmol/L | 142.0 (141-143), n=9 | 142.0 (140-143), n=7 | 0.52 |
| *Frequency above range* |  |  | *1 (11.1%)* | *0 (0.0%)* | *0.36* |
| Potassium at Baseline | 3.5-5.0 | mmol/L | 4.0 (3.9-4.4) | 4.3 (3.9-4.4) | 0.70 |
| Potassium at 1 year | 3.5-5.0 | mmol/L | 4.0 (3.9-4.4) | 4.1 (4.0-4.3) | 0.98 |
| Potassium at 2 years* | 3.5-5.0 | mmol/L | 4.3 (3.8-4.3), n=10 | 4.1 (3.9-4.2), n=12 | 0.37 |
| Potassium at 3 years* | 3.5-5.0 | mmol/L | 4.2 (3.9-4.4), n=9 | 3.8 (3.7-4.3), n=7 | 0.34 |
| Chloride at Baseline* | 99-107 | mmol/L | 104.0 (103-105), n=10 | 104.0 (103-104), n=13 | 0.49 |
| *Frequency above range* |  |  | *1 (10.0%)* | *2 (15.4%)* | *0.70* |
| Chloride at 1 year* | 99-107 | mmol/L | 103.0 (103-109), n=9 | 103.0 (101-108), n=14 | 0.54 |
| *Frequency above range* |  |  | *3 (33.3%)* | *4 (28.6%)* | *0.96* |
| *Frequency below range* |  |  | *1 (11.1%)* | *2 (14.3%)* | *0.96* |
| Chloride at 2 years* | 99-107 | mmol/L | 104.5 (103-108), n=10 | 103.5 (101-107), n=12 | 0.43 |
| *Frequency above range* |  |  | *4 (40.0%)* | *2 (16.7%)* | *0.35* |
| *Frequency below range* |  |  | *0 (0.0%)* | *1 (8.3%)* | *0.35* |
| Chloride at 3 years* | 99-107 | mmol/L | 104.0 (101-107), n=9 | 106.0 (104-106), n=7 | 0.39 |
| *Frequency above range* |  |  | *2 (22.2%)* | *1 (14.3%)* | *0.58* |
| *Frequency below range* |  |  | *1 (11.1%)* | *0 (0.0%)* | *0.58* |
| Bicarbonate at Baseline | 20-32 | mmol/L | 26.0 (23-27) | 25.5 (22-27) | 0.91 |
| *Frequency below range* |  |  | *0 (0.0%)* | *1 (7.1%)* | *0.37* |
| Bicarbonate at 1 year | 20-32 | mmol/L | 26.0 (23-26) | 24.5 (23-26) | 0.42 |
| *Frequency below range* |  |  | *0 (0.0%)* | *1 (7.1%)* | *0.37* |
| Bicarbonate at 2 years* | 20-32 | mmol/L | 24.0 (24-26), n=10 | 25.0 (21.5-27), n=12 | 0.84 |
| Bicarbonate at 3 years* | 20-32 | mmol/L | 26.0 (24-28), n=9 | 22.0 (19-24), n=7 | 0.025 |
| *Frequency below range* |  |  | *0 (0.0%)* | *2 (28.6%)* | *0.086* |
| Urea at Baseline | Male 4.0-9.0, Female 3.0-8.0 | mmol/L | 7.2 (6.0-11.2) | 8.4 (6.7-11.2) | 0.37 |
| *Frequency above range* |  |  | *3 (27.3%)* | *8 (57.1%)* | *0.14* |
| Urea at 1 year | Male 4.0-9.0, Female 3.0-8.0 | mmol/L | 6.5 (4.9-9.2) | 7.2 (4.5-10.4) | 0.55 |
| *Frequency above range* |  |  | *4 (36.4%)* | *6 (42.9%)* | *0.74* |
| Urea at 2 years* | Male 4.0-9.0, Female 3.0-8.0 | mmol/L | 7.1 (5.3-8.0), n=10 | 6.8 (4.9-11.4), n=12 | 0.77 |
| *Frequency above range* |  |  | *2 (20.0%)* | *5 (41.7%)* | *0.28* |
| Urea at 3 years* | Male 4.0-9.0, Female 3.0-8.0 | mmol/L | 7.2 (6.3-8.1), n=9 | 7.5 (7.0-15.8), n=7 | 0.15 |
| *Frequency above range* |  |  | *3 (33.3%)* | *3 (42.9%)* | *0.70* |
| eGFR at Baseline | >60 | mL/min/1.73 m^2^ | 71 (62-74) | 63 (44-83) | 0.41 |
| *Frequency below range* |  |  | *1 (9.1%)* | *6 (42.9%)* | *0.062* |
| eGFR at 1 year | >60 | mL/min/1.73 m^2^ | 72 (57-83) | 62 (45-76) | 0.26 |
| *Frequency below range* |  |  | *3 (27.3%)* | *6 (42.9%)* | *0.42* |
| eGFR at 2 years* | >60 | mL/min/1.73 m^2^ | 72 (61-81) n=10 | 60.5 (48-89) n=12 | 0.49 |
| *Frequency below range* |  |  | *1 (10.0%)* | *6 (50.0%)* | *0.045* |
| eGFR at 3 years* | >60 | mL/min/1.73 m^2^ | 65 (51-76) n=9 | 53 (31-74) n=7 | 0.19 |
| *Frequency below range* |  |  | *3 (33.3%)* | *5 (71.4%)* | *0.13* |
| Creatinine at Baseline | Male 60-105, Female 45-80 | µmol/L | 81.0 (74-91) | 90.0 (70-122) | 0.58 |
| *Frequency above range* |  |  | *4 (36.4%)* | *8 (57.1%)* | *0.31* |
| Creatinine at 1 year | Male 60-105, Female 45-80 | µmol/L | 78.0 (67-97) | 92.0 (77-104) | 0.26 |
| *Frequency above range* |  |  | *4 (36.4%)* | *8 (57.1%)* | *0.31* |
| Creatinine at 2 years* | Male 60-105, Female 45-80 | µmol/L | 83.5 (69-85) n=10 | 88.0 (65.0-111.5) n=12 | 0.32 |
| *Frequency above range* |  |  | *5 (50.0%)* | *7 (58.3%)* | *0.31* |
| Creatinine at 3 years* | Male 60-105, Female 45-80 | µmol/L | 85.0 (77-102) n=9 | 94.0 (88-154) n=7 | 0.13 |
| *Frequency above range* |  |  | *5 (55.6%)* | *5 (71.4%)* | *0.52* |

Data presented as median (IQR) for non-normally distributed continuous variables and frequency data is n (%) for categorical variables.

*Data not available for all randomised participants; n=number of participants with data included.
